# Evaluation of the Mechanical Properties of Pine, Larch, and Spruce Wood Subjected to Saline Treatment

**DOI:** 10.3390/ma19061108

**Published:** 2026-03-12

**Authors:** Kamil Roman, Emilia Grzegorzewska, Mateusz Leszczyński, Seweryn Pycka, Negin Hamidi

**Affiliations:** 1 Department of Technology and Entrepreneurship in the Wood Industry, Warsaw University of Life Sciences (SGGW), 166 Nowoursynowska Str, 02787 Warsaw, Poland; kamil_roman@sggw.edu.pl (K.R.); emilia_grzegorzewska@sggw.edu.pl (E.G.); mateusz_leszczynski@sggw.edu.pl (M.L.); seweryn_pycka@sggw.edu.pl (S.P.); 2 Department of Food Biotechnology and Microbiology, Warsaw University of Life Sciences (SGGW), 166 Nowoursynowska Str, 02787 Warsaw, Poland

**Keywords:** wood tension, stresses, salted water soaking, tensile strength along fibers, coniferous trees

## Abstract

**Highlights:**

- An energy-based method was applied to characterize tensile deformation of saline-conditioned coniferous wood.
- Tensile behavior was quantified by measuring total tensile work from stress–strain relationships.
- Specimens with comparable tensile strength exhibited distinct energy demand and deformation behavior.
- Saline conditioning influenced deformation mechanisms and energy absorption beyond the effects of strength alone.
- The proposed approach enables improved assessment of deformation tolerance and failure behavior under variable environmental conditions.

**Abstract:**

Structures made of wood are used extensively in applications that require mechanical reliability under variable environmental conditions. Several softwood species were investigated, including pine (*Pinus sylvestris* L.), spruce (*Picea abies*), and larch (*Larix decidua*). This study investigated the tensile deformation behavior of each species with a special focus on the mechanical energy demand of the tensile process. Samples were conditioned in an aqueous saline medium for defined exposure periods and compared with controls. The energy of deformation was determined from stress–strain relationships of tensile tests under identical loading conditions. Results indicate that saline conditioning alters the tensile response of the examined wood species in a species-dependent way. Tensile strength increased in pine wood after exposure, whereas spruce and larch showed different trends depending on conditioning duration. A wide range of tensile strengths was recorded for all samples, ranging from 5.4 MPa to 102.0 MPa. Controlled saline exposure significantly influences the mechanical behavior of softwood species, as indicated by the findings. Evaluating wood performance under modified environmental conditions, both deformation energy and strength parameters should be considered. The main novelty of this study is the introduction of an energy-based description of tensile deformation, in which the total tensile work is calculated from force–displacement relationships, enabling differentiation of specimens with similar tensile strengths but fundamentally different deformation and failure properties. A practical advantage of the proposed energy-based approach is that it provides additional insight into the deformation tolerance and failure behavior of saline-conditioned wood, thus enabling a more reliable assessment of material performance under unpredictable environmental conditions.

## 1. Introduction

Since ancient times, wood has been utilized in shipbuilding and structural applications; however, its properties and performance remain strongly dependent on the conditions under which it is exposed [1,2,3,4]. Despite its widespread use in wooden structures, coniferous wood is susceptible to fungal degradation in humid environments [5]. To maintain service life and functional reliability, protective treatments are generally necessary [6,7]. The environment can also influence the development of deformation mechanisms and strength mechanisms, particularly under sustained or cyclic mechanical loads [2,6,7]. Wood performance is influenced primarily by moisture content. A key factor influencing wood behavior is its moisture content. The presence of water or high relative humidity can lead to a loss of structural integrity in coniferous species [5]. Because wood is hygroscopic, variations in moisture content lead to swelling and shrinkage, which directly affect its strength and dimensional stability [8,9,10]. Solid wood may warp, crack, and deform due to shrinkage and swelling, limiting its use in load-bearing elements. The degree of instability also affects the amount of mechanical energy required to deform the material under tensile loading [2].

Internal stress redistribution contributes to these dimensional changes, which, in turn, affect the material’s tensile behavior. The wood industry often uses controlled protective conditions to limit biological degradation, as fungi and insects cannot develop outside of specific moisture ranges [3,11]. Physical factors, including cyclic moisture variation, mechanical load, and ultraviolet radiation, also influence the degradation of an object. Deformation, cracking, and the release of internal stress occur due to repeated swelling and shrinkage [12,13], thereby increasing permeability and further degrading the material. Mechanical performance is therefore affected by maintaining appropriate moisture conditions, particularly in applications exposed to long-term environmental variability [14,15]. Wood can be mechanically modified, chemically coated, thermally treated, or impregnated to reduce moisture-induced degradation. To limit moisture transport, reduce internal stress development, and improve the mechanical reliability of wood under variable environmental conditions, mechanical modification, surface coating, chemical treatment, thermal modification, and impregnating methods are commonly employed [16]. It has been shown that the use of appropriate chemicals can enhance the stiffness of cell wall structures by modifying fiber–matrix interactions, reducing microfibril mobility, and improving stress transfer within the cell wall [17], confirming that energy-based approaches to mechanical behavior are relevant. Chemical treatments based on salts have a long history of controlling moisture-related effects. Sodium chloride is one of the traditional salt-based treatments used for centuries to modify wood, improving its durability and resistance to environmental factors.

Mechanical loading causes cellular and subcellular changes that influence stiffness, strength, and energy dissipation. The physical and mechanical properties of unprotected wood decrease over time due to progressive degradation [18,19]. Depending on the mechanical properties, even moderate changes may have significant safety implications [20]. Previous research has examined the influence of moisture content and saline environments on the mechanical properties of coniferous wood, with particular emphasis on strength-related parameters. Numerous studies have shown that exposure to seawater or salt solutions alters tensile strength and stiffness in a species-dependent manner. Earlier investigations within a systematic research cycle demonstrated that pine, spruce, and larch exhibit distinct tensile-strength responses following controlled seawater conditioning [3,4]. Similar changes in mechanical performance have also been reported for long-term salt-exposed wood and were attributed to moisture-induced degradation and salt–cell wall interactions [5]. The selection of Scots pine, Norway spruce, and European larch enables analysis of mechanical behavior across a broad spectrum of softwoods, from relatively low-density pine and spruce to denser, more compact larch. During loading, tensile deformation behavior, fracture mechanisms, and energy absorption will differ among these species due to their densities, cell structures, and resistance to environmental factors.

These studies have predominantly focused on maximum tensile strength, apparent stiffness, or elastic parameters, which do not capture the full deformation and failure process associated with tensile loading. In particular, the energetic aspects of tensile deformation of saline-conditioned coniferous wood, such as the total mechanical work derived from stress–strain relationships, have not been systematically investigated. The energy-based characterization of tensile deformation of timber exposed to saline environments remains a clear research gap, which this study addresses by applying controlled NaCl conditioning to pine, spruce, and larch wood and introducing an energy-based description of tensile deformation. A significant benefit of this approach is that it allows us to evaluate the deformation mechanisms, energy absorption, and failure behavior of saline-conditioned wood beyond its tensile strength alone [21,22,23]. Using an energy-based approach, this study examines the tensile deformation behavior of saline-conditioned coniferous wood. An additional descriptor to conventional strength-based parameters in this study is total tensile work derived from stress-normalized displacement relationships.

## 2. Materials and Methods

### 2.1. Study Material

The study used Scots pine (*Pinus sylvestris* L.), Norway spruce (*Picea abies*), and European larch (*Larix decidua*) wood. The wood was harvested from forests in Poland that grow naturally in similar habitats and climates. Material was obtained from nine mature trees, with three trunks representing each species. The trees were approximately 80 years old. At a height of 1–3 m above ground level, logs were collected from the main stem to minimize longitudinal variation. The material homogeneity and stability of the specimens were enhanced by excluding sapwood from all specimens. All species were selected according to the same criteria. Experimental conditions were the same for all specimens made from construction-grade lumber. Allows for distinguishing material-dependent effects from those associated with wood as a natural composite material. Managed forests in Poland provide wood material, which is characterized by a temperate climate and moderate rainfall [24].

Construction and wood-based industries typically use coniferous species under such conditions. Grass-fed timber originates from mature trees, ensuring full anatomical development and mechanical stability. Selection of materials accounts for the inherent anisotropy of wood and the need to minimize variability unrelated to species-specific behavior. As a result, heartwood is considered, since it is commonly used for structural purposes and exhibits more stable physical and mechanical properties. It is necessary to conduct a visual inspection to detect defects such as knots, cracks, fiber deviations, and resin pockets that can impact the mechanical performance of the part. The material can be classified in the standard construction quality group, despite minor surface imperfections that do not compromise its structural integrity [25].

Comparing pine, spruce, and larch provides a meaningful comparison of different materials with varying densities, cellular structures, and natural resistance to environmental factors. Spruce is valued for its relatively uniform texture and elastic response, while larch stands out with higher density and natural durability. Compared with other wood types, pine exhibits a favorable strength-to-weight ratio and good processing properties. Differential tensile deformation behavior, fracture mechanisms, and failure energy are expected to be affected by species-specific characteristics. Results are relevant to real engineering conditions, as the wood species commonly used in structural applications are included. A comparative assessment of tensile behavior and energy demand across wood species is possible because of the anisotropic nature of wood, which involves complex deformation and energy-absorption mechanisms.

### 2.2. The Material NaCl Treatment

**Figure 1:**
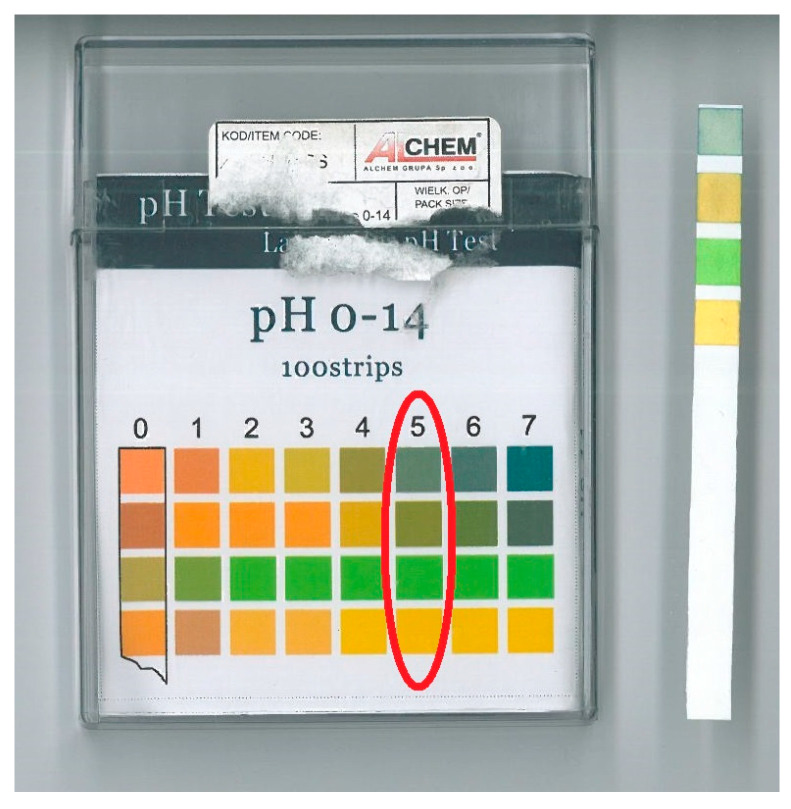
The pH result of the solution measurement.

Research was conducted to determine the energy required to measure the durability of wood samples modified in medium-salinity water solutions. This study was based on a previous analysis published in the literature [3,4,26]. The test required the preparation of standardized wood samples from Scots pine (*Pinus sylvestris* L.), European Larch (*Larix decidua*), and Norway spruce (*Picea abies*). A total of 120 samples were tested for each wood species. The tests involved placing standardized samples in a cuvette containing NaCl solution, followed by tensile tests. With distilled water and sea salt, 60 L of water were poured into a basin containing the specimens. To prepare the NaCl solution, 7 g of salt was dissolved in 1000 g of water at the correct ratio [3,4,5]. The salinity of 7‰ NaCl is the same as that of the Polish Baltic Sea. The samples were immersed in salt water in each cycle and then removed from the container as required. Samples underwent four conditioning stages: one untreated reference stage and three saline-soaking cycles (*I*–*III*). In cycles *I*, *II*, and *III*, the samples were soaked in a NaCl solution for 2, 4, and 6 weeks, respectively. The pH of the prepared solution was measured as a control. The pH result is presented in Figure 1.

As a result of the study, the properties of the wood were compared to those of the native material after NaCl cycling. The duration of the saline exposure was 2, 4, and 6 weeks for cycles *I*, *II*, and *III*, respectively. The wood should be soaked in an unsaturated salt solution. This reduces the chances of crystals forming on the wood surface. The sample’s weight was used to estimate the amount of liquid absorbed during immersion. According to the test methodology [3,4], the samples were removed from the container and dried at 103 °C in a laboratory chamber. The samples should be prepared according to the required moisture content before the strength test [27]. To standardize the moisture content of all samples, the samples were placed in a conditioned chamber for an extended period. The sample moisture content remained at 12% after two weeks [3,4]. The procedure for measuring moisture content was repeated each time before testing to determine the results. The samples were stored in airtight containers to ensure proper storage and quality control. The procedure of saline exposure can be summarized into four stages. These stages include the treatment duration and experimental conditions.

**Figure 2:**
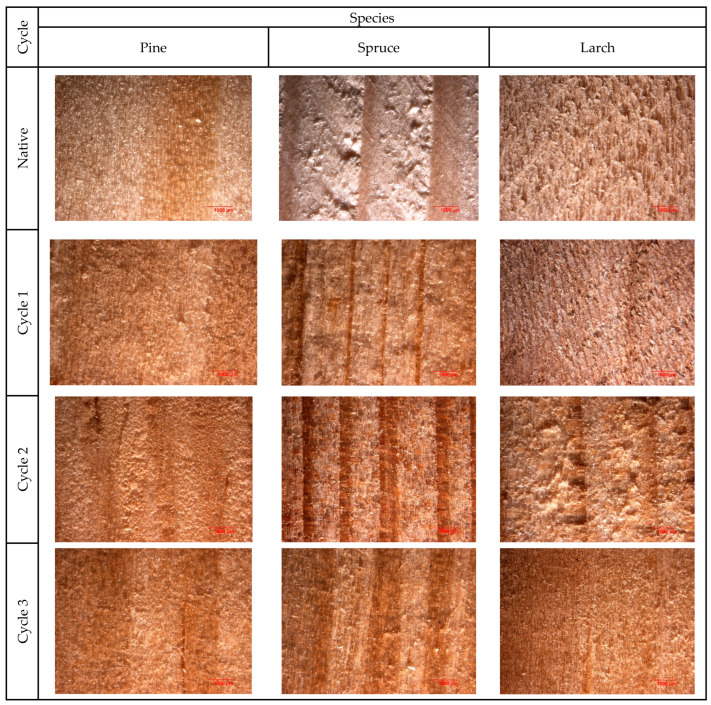
Optical micrographs of wood sample surfaces for each species (pine, spruce, and larch): native (control) condition and after saline treatment cycles *I*–*III*.

After each saline-conditioning cycle, the samples were removed from the solution and oven-dried at 103 ± 2 °C until constant mass was achieved. Subsequently, all specimens were conditioned in a climate chamber under controlled environmental conditions until equilibrium moisture content was reached. Before mechanical testing, the moisture content of all samples was stabilized at 12%, in accordance with standard reference conditions for wood testing. Sample mass was monitored during conditioning, and weighing was performed only after stabilization, ensuring identical moisture conditions for all tested variants. This procedure eliminated the influence of moisture variability and enabled reliable comparison of tensile strength, deformation behavior, and energy-related parameters among species and treatment cycles. These qualitative observations are illustrated by representative optical micrographs shown in Figure 2.

The effects of saline conditioning on wood tissues were evaluated qualitatively using optical microscopy. Before mechanical testing, observations were focused on anatomical features and surface morphology. A general structural modification associated with salt exposure is illustrated in the micrographs. A fracture-zone microstructural analysis of failed specimens was not conducted, as the main objective of the study was to assess mechanical response and energy-based parameters rather than to detail fracture mechanisms. As the study focused on mechanical performance rather than transport or diffusion, the depth of salt solution penetration into the wood structure was not quantified.

### 2.3. Study Material Color Testing

**Figure 3:**
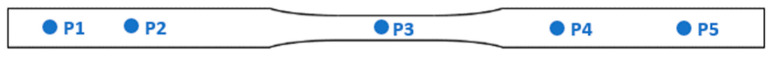
Wood sample with marked measurement points used to determine color parameters in the colorimetric tests.

Scots pine (*Pinus sylvestris* L.), European larch (*Larix decidua*), and Norway spruce (*Picea abies*) were calorimetrically characterized in the CIE L*a*b* color space using a spectrophotometer (Erichsen 565, Erichsen GmbH, Hemer, Germany). The color measurements were performed on tangential surfaces because this anatomical plane is sensitive to surface structure, growth-ring arrangement, and moisture changes, and allows comparisons across species with different anatomical characteristics. Lightness, red-green chromaticity, and yellow-blue chromaticity were used to describe surface color. The parameters were treated as independent variables and reported as separate columns in the results tables, which allows direct comparisons among wood species and conditioning variants. The color and gloss were measured on 30 samples of each wood species, resulting in 120 samples in total. On each specimen, measurements were performed at five points on the tangential surface. Figure 3 shows a wood sample with marked measurement points used to determine color parameters in the colorimetric tests.

The reported values represent the arithmetic mean of the measurements taken at these points. Color measurements were conducted on each sample at five predefined points on the tangential surface, located at the corners of the rectangular surface and at the geometric center, to reduce the influence of local anatomical variation. Color differences between reference and conditioned samples are quantified using the total color difference parameter (Δ*E*), which represents the Euclidean distance between two points in the three-dimensional L*a*b* color space. Lightness (L*), chromatic coordinates (a*, b*), total color difference (Δ*E*), and gloss (*G*) were recorded for each specimen. The E value integrates lightness and chromatic coordinates, providing a method for objectively evaluating the intensities of surface modifications. According to the following relationship, the total color difference is calculated:

$$∆E=\sqrt{{\left({∆L}^{*}\right)}^{2}+{\left({∆a}^{*}\right)}^{2}+{\left({∆b}^{*}\right)}^{2}}$$

Colorimetric assessment is enhanced by analyzing surface gloss (G) as a complementary optical parameter, providing additional information on visual appearance and surface reflectivity. Depending on the wood species, gloss values are affected by surface roughness, fiber orientation, and anatomical heterogeneity. Methodological consistency is ensured by measuring the same tangential surfaces as the color parameters. Representative values are obtained by averaging multiple measurements at each specimen. In the result, gloss appears alongside L*, a*, b*, and Δ*E*.

### 2.4. Tensile Testing

Using the Instron 3382 universal testing machine (Norwood, MA, USA) with suction-type tensile grips, tensile testing is performed along the fiber direction. Instron IX software controls the measurement workstation, which includes a measuring machine, tensile clamps, and an auxiliary holding system. Material behavior can be characterized through tensile testing under uniaxial loading conditions [3,28]. As part of this framework, tensile loading provides insight into the tensile strength and deformation behavior of steel via stress–strain relationships. The mechanical energy associated with the tensile deformation process can then be analyzed.

The tensile tests follow ISO 527-1 [11], ensuring the repeatability and comparability of results across different materials and conditioning scenarios. To maintain consistent loading conditions across all specimens, reliable comparisons of mechanical responses can be made. Using the acquired data, it is possible to evaluate differences in tensile strength, deformation behavior, and energy consumption during loading between wood species and treatment variants. The tensile test results are statistically analyzed using analysis of variance (ANOVA) [29,30,31]. To identify statistically homogeneous groups among the tested samples, Duncan’s multiple-range post hoc test is used. Based on this statistical approach, differences in mechanical response, including parameters related to deformation energy, can be interpreted. Using stress–strain data to calculate total tensile work is a key methodological contribution.

Based on the force and crosshead displacement data, engineering stress–strain curves were used to characterize specimen tensile behavior. An extensometer was not available during testing to measure local strain directly. The crosshead displacement was normalized with respect to the gauge length of the specimen to obtain a relative deformation parameter. Deformations can be expressed in dimensionless forms with this approach, which allows consistent comparisons of tensile behavior between identical specimens. The engineering strain was calculated based on the normalized crosshead displacement relative to the initial gauge length, while engineering stress was calculated based on the applied force and the initial specimen cross-sectional area. An engineering strain is represented by the horizontal axis of the curves, and an engineering stress by the vertical axis. Normalized deformation derived from crosshead displacement provides a reliable and repeatable method of measuring strain. Based on the obtained stress–strain curves, the materials’ tensile behavior, including their deformation behavior and mechanical performance, was analyzed.

## 3. Results

### 3.1. Analyzing the Study Material Color

Three softwood species were analyzed using quantitative colorimetric and gloss parameters, including Scots pine (*Pinus sylvestris* L.), European larch (*Larix decidua*), and Norway spruce (*Picea abies*). The evaluation focused on tangential surfaces, which reflect geometrical features and optical properties of each species. Color characteristics are described by the CIE L*a*b* system, where L* represents lightness, a* corresponds to the green–red chromatic axis, and b* describes the blue–yellow chromatic axis. The surface gloss (*G*) is included as an additional element to complement the assessment of visual appearance and surface reflectivity. The measured values of L*, a*, b*, and G are presented as separate parameters. The wood species and reference materials can be directly compared. Based on the tensile tests performed along the fiber directions on the native materials discussed in this subsection. A summary of the baseline color and gloss characteristics is presented in Table 1.

Table 2 shows the mean total color difference (ΔE) and standard deviation (SD) for three wood species after successive cycles of saline treatment. The values represent averages calculated from 120 samples per species and cycle.

The visual and optical properties of softwood species can be characterized using colorimetric and gloss parameters. The surface gloss, lightness, and chromatic coordinates of Scots pine, European larch, and Norway spruce differ significantly, reflecting species-specific anatomical structures and chemical compositions. Lightness values were highest in European larch, while Scots pine had a darker appearance and a more pronounced red chromatic component. Norway spruce color properties exhibit intermediate characteristics with a slightly enhanced yellow component. Across all native materials, gloss values were low, indicating diffuse light reflection typical of untreated tangential wood surfaces. By comparing these baseline color and gloss characteristics with mechanically or chemically modified materials, further analyses can be conducted to help interpret subsequent changes in optical behavior.

### 3.2. Analysis of the Tensile Process

The results of the tensile test show that different wood species undergo varying degrees of change. Tensile properties of wood are thought to be differentiated because of the anisotropic structure. For each species, distortions and nonlinearity were observed during the static tensile test. Cracks in the anisotropic structure of the material might be responsible for this disturbance. Furthermore, the anisotropic structure of wood may affect its mechanical properties. To better understand how wood structure affects the tensile process, studies are conducted. Throughout the testing, the grip of the testing machine moved at a constant speed of 5 μm per second. A species of wood should change its force as a function of normalized displacement, just as the quality of the wood changes because of normalized displacement. The energy required to tear a specimen is determined by the size of the hole formed during the tearing process.

#### 3.2.1. Pine Wood Samples

**Figure 4:**
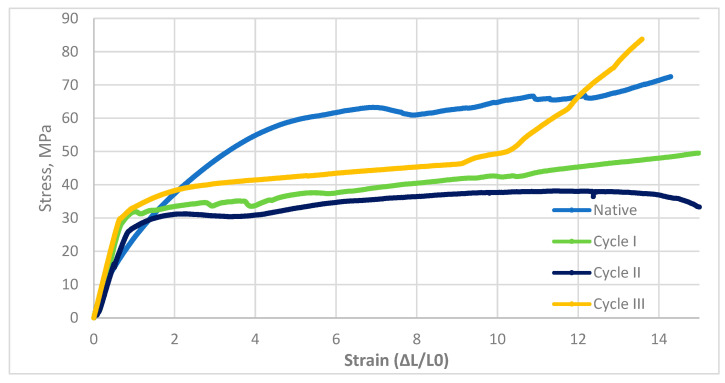
Stress–strain response of pine wood samples subjected to saline treatment.

The curves represent mean tensile responses, and the low scatter among individual stress–strain records confirms the homogeneity of the results. The characteristics of a tensile process for pine wood are shown in Figure 4.

The static tensile test was considered complete upon fracture. The above diagram shows that the tensile response of the analyzed material varied with the soaking cycle. The presented curves represent the mean responses of the tested specimens, and the low variability among individual measurements verified the homogeneity of the results. Due to the material’s anisotropy, the initial stage of loading was critical. Differences in fracture behavior were observed with increasing number of modification cycles, with the native and cycle *III* samples exhibiting the lowest normalized displacement values. In the early stages of the process, an increase in stress with applied strain was observed; however, despite the apparent proportionality, yield strength was difficult to determine because of material anisotropy.

The experiment showed that wood pine properties changed under different loading cycles, compared with other materials, demonstrating that wood pine is highly variable. The apparent stiffness parameter of pine stretching can be calculated as the trend line adjacent to the function, whose coefficient of determination R^2^ gives the deviation value from the predicted function. According to the apparent stiffness parameter, the material has moderate stiffness, and the coefficient of determination indicates some agreement with the theoretical model. An increase in stiffness is likely to be accompanied by a doubling of the apparent stiffness parameter in cycle *I*. It suggests that the model fits well when the R^2^ coefficient is high. In cycle *II*, E represents the apparent stiffness parameter, and the coefficient of determination was high. The evident stiffness parameter reached its highest value in cycle *III*, accompanied by an equally high R^2^. The results indicate a significant impact of loading cycles on several mechanical properties of pine wood. The values of maximum strain and yield strength for pine wood are shown in Table 3.

#### 3.2.2. Larch Wood Samples

**Figure 5:**
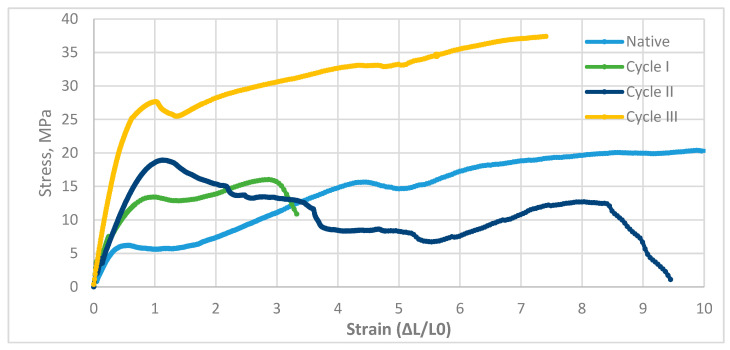
Stress–strain response of larch wood samples subjected to saline treatment.

The static tensile test for Larch wood confirmed the lack of linearity observed in each cycle. Stretching of Larch wood was characterized by significant variations in stress. The native cycle and cycle *II*, in which the specimen failed, showed that the stress had nearly fallen to zero before failure. This suggests that the Larch wood samples had an elastic limit below which the stress was significantly reduced. Larch wood has an elastic limit, which determines its ability to resist stress. This behavior was unexpected and was not observed in other Larch wood samples. There may be an anisotropic structure in the wood, which explains the varying course of tensile behavior. The material’s anisotropic structure may cause deviations from linearity in the graph. Other wood species have experienced similar situations in the past. Depending on the soaking cycle, Larch wood samples are expected to exhibit characteristic changes in stress–strain. There was relatively low scatter in individual stress–strain records, confirming the homogeneity of the results during the course of each treatment cycle based on the presented curves. The tensile characteristics of Larch wood are shown in Figure 5.

The diagram shows that the tensile process for the examined materials varied according to the soaking cycle they received during the tensile test. It was found that during the early stages of the process, the resulting stresses in the material increased with the applied strains. This suggests that the material was becoming more elastic and ductile, allowing it to stretch further without causing permanent damage. Based on the study, wood Larch properties differed across loading cycles, unlike those of other materials, demonstrating that wood pine properties are highly variable. The material apparent stiffness parameter indicated moderate stiffness, and the coefficient of determination indicated some agreement between the theoretical model and the measurements. The results indicate that loading cycles impact several mechanical properties of Larch wood. The maximum elasticity, normalized displacement, and yield strength of Larch wood are presented in Table 4.

#### 3.2.3. Spruce Wood Samples

**Figure 6:**
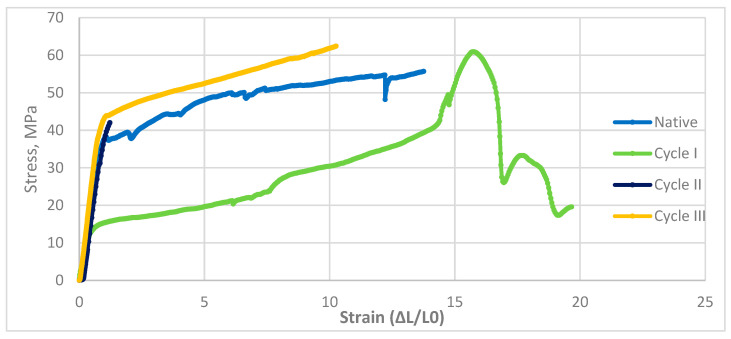
Stress–strain response of spruce wood samples subjected to saline treatment.

In the case of the static tensile test of spruce wood, it was also observed at the beginning of the process that partial linearity was not observed at the start of the test. The disturbance in linearity is evident in the value of the determination coefficient R^2^ for natives and cycle *I*, particularly when there is a disruption in linearity, as seen in the case of natives and cycle *I*. The aforementioned factors may cause the crack that appears during the process. There was also significant variation in the amount of stress applied during the remainder of the Larch wood stretching process. There was a considerable amount of variability, especially in Cycle *I*, which was readily apparent. Multiple factors, rather than one specific factor, are likely to have contributed to the crack. Moreover, the effects of this variation on the mechanical properties of spruce wood may also be significant in terms of these changes in the wood’s properties. Wood may exhibit variable tensile behavior due to its anisotropic structure, microporosity, and other factors. As a result of this variability, wood may be less intense, stiffer, and tougher than it would be otherwise. In addition to its mechanical properties, wood’s performance can also be influenced by its inherent variability. The stress increased linearly after exceeding the yield point in the native case and cycle *III*, but at large deformations. The typical brittle structure of Cycle *II* persisted and cracked as a result. The tensile strength characteristics of spruce wood are shown in Figure 6.

The graph clearly shows that one of the courses is more irregular than the other. The presented curves represent mean tensile responses, while the low scatter among individual stress–strain records confirmed that the force increase was similar in the first stage. There was an increase in force in each case due to a rapid increase in stress at the beginning of the process. This stress increased gradually as it exceeded the proportionality limit, whereas deformation increased slowly. The material became increasingly flexible over time. The material was stiffer at the beginning of the process, so it could withstand less stress, leading to a more rapid increase in force. When the material became more flexible, it was better able to absorb stress, allowing its shape to change more slowly as stress increased. Although the yield strength appeared to be proportional, anisotropy made it challenging to calculate in each case study. There has been evidence that the properties of spruce change with the amount and duration of soaking. The apparent stiffness parameter indicated moderate stiffness, and the coefficient of determination showed some agreement between the theoretical model and the measurements. The test results suggest that soaking cycles alter the mechanical properties of spruce wood. The maximum elastic normalized displacements and yield strength of the spruce wood are presented in Table 5.

Yield stress was defined as the stress at which the first visible surface crack appeared during tensile loading and is reported in the tables. The research on pine, Larch, and spruce was conducted across three species. To analyze the results, we provide information on the cycle of soaking the material in the NaCl solution, including the apparent stiffness parameter, the coefficient of determination (R^2^), and the maximum elastic deformation and tensile strength for each sample. Considering these values, the mechanical properties of wood can be analyzed based on wood species and the duration of material soaking. Comparing the tensile strength of pine in cycle *III* to that of other wood species and cycles, the tensile strength of pine is reasonably low by comparison. Compared with its predecessors, Larch wood exhibits a larger maximum elastic deformation in cycle *II*. According to these results, Larch wood exhibits higher mechanical properties than pine wood, and the duration of the soaking process significantly influences these properties.

#### 3.2.4. Failure Mechanisms

An engineering stress–strain curve was used to characterize tensile behavior and compare mechanical performance of the specimens. Stress was calculated from the applied force and the initial cross-sectional area of each specimen, ensuring that specimen geometry was normalized. To calculate engineering strain, the crosshead displacement was normalized by the gauge length at the start. The strain values were directly calculated from machine-recorded displacements. The procedure ensured repeatable testing conditions and allowed comparison of tensile responses across specimens despite differences in displacement values. Based on the morphology of the stress–strain curves, particularly in the post-peak region, it was possible to identify the dominant failure mechanisms in the examined wood species. After reaching maximum stress in pine wood, the stress gradually decreased, indicating progressive fiber rupture and stable crack propagation before final failure. Cycle *II* samples, however, showed a pronounced and rapid stress drop after the peak, indicating a more brittle fracture mode. Post-peak behavior reflects variations in energy absorption, as reflected in the calculation of tensile work.

The stress–strain trajectories of larch wood are more irregular, especially in cycle *II*, when local stress reductions occur before ultimate fracture. During loading, this pattern suggests crack initiation within the anisotropic cellular structure and unstable crack propagation. Compared to cycle *I* specimens, native specimens showed a smoother stress evolution and more evenly distributed damage before rupture. Variability in curve shape confirms that larch’s tensile response is highly sensitive to conditioning duration and structural heterogeneity. The fracture behavior of spruce wood was clearly cycle-dependent. Deformation beyond the proportional limit of native and cycle *III* specimens was relatively stable, suggesting fiber separation before failure and a higher deformation tolerance. Cycle *I* and *II* samples, however, were characterized by increased apparent stiffness with a rapid decline in stress, which is typical of brittle fractures with limited post-yield deformations. Stress–strain analysis shows that saline conditioning alters both tensile strength and stiffness, as well as fracture mode and deformation energetics, as evidenced by differences in total tensile work.

### 3.3. The Tensile Process Energy Consumption

Compared to earlier tensile studies on saline-treated wood that focused solely on maximum strength values [3,4], this study provides additional information. The tensile work was calculated by integrating the stress–strain relationship during static tensile loading. As a result of this energy-based parameter, the entire deformation process from the beginning to the end can be captured, including the elastic and inelastic stages and the redistribution of internal stresses within the material. Similar specimens with similar tensile strength but fundamentally different deformation and failure behaviors can be differentiated from one another. The normalized displacement data were recorded in millimetres, while the polynomial fit variable was expressed in metres; therefore, the integration upper limit was 0.001 mm.

As a model estimation method, the Gauss–Newton least-squares method was employed, ensuring that the coefficient of determination (R^2^) was greater than 0.9 throughout the entire estimation procedure. To ensure the model fits the obtained data, the procedure was designed to achieve sufficient accuracy. It was found that the third-degree polynomial provided a good fit to the described arithmetic function, which accurately describes the model stretching process of wood from different species in most cases. Various degrees of polynomials (from 2 to 6) were applied according to the goodness of fit (R^2^). The best fit for Spruce Cycle *II* was obtained with a second-degree polynomial. The integral equation of the total tensile work is presented in Table 6.

The determination coefficient indicates the validity of using a third-degree polynomial to model the forces during the process, thereby facilitating understanding of the changes. In accordance with the methodology for selecting the polynomial based on the determination coefficient, the fit of the function defining the compaction process improved. In Cycle *I*, spruce was modified in accordance with the Cycle *I* specifications. In one case, the R^2^ remains below 0.9 despite a sixth-degree polynomial; in another, it is 0.8701. However, a trend line was still fitted with a second-degree polynomial if the R^2^ coefficient was 0.900 or greater. Based on variations in compaction force over time for different raw materials and types of modification, the total work of the compaction process for each material was calculated.

The higher total tensile work values observed for pine wood in cycles *I* and *II*, compared to spruce and larch, are attributable to species-specific deformation mechanisms rather than to strength alone. Pine wood is characterized by lower density, higher microfibril angle variability, and a more compliant cell wall structure. Short- and medium-term saline conditioning may increase moisture-induced plasticization and redistribute internal stresses within cell walls, thereby increasing deformation capacity before fracture. As a result, pine specimens subjected to cycles *I* and *II* exhibited larger normalized displacements at comparable force levels, which directly translated into higher total tensile work. In contrast, spruce and larch, with denser and more rigid anatomical structures, exhibited more brittle, strength-dominated failure behavior, limiting normalized displacement and reducing energy absorption. Therefore, the elevated mechanical work values for pine in early conditioning cycles reflect enhanced deformation tolerance rather than increased tensile strength.

The results of the study showed that the multinomial model fitted to the trend line closely correlated with the total work required for material compaction. Due to the multinomial nature of the model, it can accurately predict the effort to be invested across the entire process.

### 3.4. Strength Testing

Based on the static tensile test results, they comply with the current standard. For the prepared samples, the tensile process was conducted at 22 °C in the laboratory. To determine how much the samples deformed during the tensile process, the forces applied to them in special holders were measured. To calculate the material’s tensile strength, the test results were used. Calculating the tensile strength of a sample involves dividing its cross-sectional area by its maximum load. To determine the material’s quality, its tensile strength was compared with the current standard. The pressure along the fibers is expected to be higher during the stretch process than when the fibers are arranged transversely, as they are then stretched along their length. Although anisotropic materials cracked when pushed, they still had high stress values. Therefore, it was decided to introduce a yield stress, measured to the first surface crack. The tensile tests performed on pine, spruce, and Larch samples in native (non-soaked) material are presented in Table 7.

**Figure 7:**
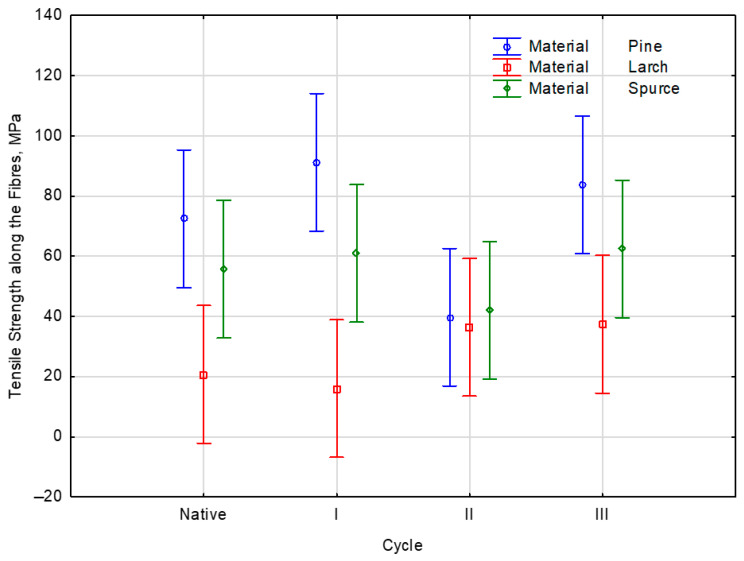
The number of soaking cycles in salted water affects the tensile strength of wood species.

Each test cycle was conducted along the length of the fibers to determine their tensile strength. Further statistical analysis revealed that the number of soaking cycles influences the tensile strength of the different wood species. As the number of soaking cycles increased, the tensile strength decreased. In some species, the decline was more marked. Statistical analysis revealed that species with the highest tensile strengths showed the slightest decrease in tensile strength with increasing cycles. Based on the statistical analysis, there was significant variation in the measured parameters and a correlation between them. Considering the correlation among the analyzed indicators, there was no significant influence on the correlations, as confirmed by the *p*-value of 0.131. The empirical value of *F*(6, 24) = 1.854 was consistent with the *p*-value of 0.131. The averages of the analysis of the influence of the number of applications of soaking cycles in salted water on the tensile strength values of a given wood species were presented in Figure 7.

Pine, Larch, and spruce wood showed different effects across individual samples, as determined by the post hoc Duncan test. The statistical analysis of the strength parameters of the samples from the analyzed wood species showed a lower degree of significance than the accepted level of significance, α = 0.05. Therefore, the average effects of soaking cycles on tensile strength differ when analyzed by cycle number. A homogeneous group of individuals is formed by the three kinds of wood analyzed: Pine, Larch, and Spruce. The tensile strength parameters of the prepared samples were previously studied by soaking them in a sodium chloride (NaCl) solution. In this case, the degree of significance (*p*) was higher than the acceptable alpha significance level of 0.05, indicating that the result was not statistically significant at this time. The tensile strength parameters of the samples, measured across successive soaking cycles, were statistically analyzed to determine their effects on the test wood’s parameters. Regardless of the material type, the sample’s strength remained relatively unchanged with increasing cycles. According to the post hoc test, only one univariate group showed low-square averages for the soaking scores for each assailant.

## 4. Discussion

Unlike earlier studies on saline-treated coniferous wood that focused solely on tensile strength [3,4], the present analysis reveals differences in deformation behavior that cannot be captured by strength parameters alone. The tensile strength of coniferous wood treated with seawater has been reported to vary considerably across species and treatment duration in earlier studies [3,4]. According to the present studies, specimens with similar tensile strengths differ significantly in tensile work but exhibit similar tensile strengths. The influence of saline conditioning on wood’s mechanical behavior can be underestimated by strength-based evaluation alone. Previous strength-centric studies focused purely on tensile strength could not detect such deformations and failures [3,4]. According to the applied energy-based approach, these characteristics have been detected. The physical properties of the wood depend on the type of wood that has been soaked. There may be differences among timber species in density, porosity, and moisture content. These parameters make wood strong, stable, and durable [18,32,33].

Some important conclusions can be drawn from analyzing test results on the mechanical properties of different types of wood [22,23,34]. Native pine wood has a relatively compliant structure, which can be attributed to its lower density and greater microfibril mobility. The presence of salt appears to modify this response by preventing cell wall deformation, thereby increasing stiffness. Treating pine wood can improve its mechanical properties, as the values are higher for pine *I*, *II*, and *III*. Moreover, the tensile strength of so-pine *III* (83.73 MPa) along the fiber direction increased, which may be beneficial for structural applications, such as wind turbines. Similarly, after six weeks, a significant increase in strength can be observed, though it is not immediate. Based on the results, it can be concluded that pine, like similar species that are also po-positive in seawater, is also po-positive in freshwater.

As part of the testing process, Larch wood will be tested next. Compared with the other species tested, larch exhibits relatively elastic deformation behavior, as indicated by its apparent stiffness parameter. These values also increase in Larch cycles *I*, *II*, and *III*, which can be attributed to the beneficial effect of the treatment process on the wood’s mechanical properties. The tensile strength of Larch cycle *III*, the strongest wood species used in this study, was 46.473 MPa along its fibers. Based on the analyses, the smallest strength difference was observed between the arch and the other woods. Larch wood, as well as various species of wood, exhibited an increase in strength only when samples were soaked for a period of four to six weeks before testing.

According to the apparent stiffness trend observed for Spruce cycle *I*, *II*, and *III*, the apparent stiffness parameter of the wood is significantly higher than that of the wood when it is in its natural state. The mechanical properties of spruce wood improve more after treatment, making it more likely to enhance its mechanical properties. In comparison to pine and larch, spruce exhibits the most dramatic strengthening response following prolonged saline exposure, suggesting that salt ions interact more strongly with its cell wall structure. The study’s results showed that wood treatment processes significantly improved the mechanical properties of wood, particularly its apparent stiffness parameter and tensile strength. The ratings for each type of wood increase with the number of treatment cycles, making it easy to determine if a material is suitable for a particular application based on its characteristics. According to these results, saline conditioning alters not only tensile strength but also stiffness-related responses, potentially affecting structural reliability [22].

Mechanical responses discussed above can also be interpreted by considering the optical characteristics of the tested materials and energy-based descriptions of deformation. Colorimetric parameters determined for native wood species reflect differences in chemical composition, extractive content, and surface structure, all of which have been reported in the literature to affect moisture interaction and mechanical performance. Lightness and changes in chromatic coordinates are often associated with the redistribution of extractives and lignin-related compounds near the surface of a material, which can, in turn, indirectly affect gradients stiffness and stress transfer under load [14]. As a result of the integrated description of total tensile work, wood treatment may influence not only maximum strength parameters but also the material’s ability to absorb energy during deformation. The viscoelastic behavior of wood materials after conditioning is modified by altered surface coloration and increased chromatic saturation, even when the changes in tensile strength remain moderate. According to the observed color characteristics and variations in tensile work, surface-related chemical and physical modifications influence the mechanical response of treated wood [23].

The tensile strength results indicate that, in some instances, there is a slight improvement in Larch species. Following successive soaking cycles, the samples’ strength parameters will improve, as shown in the graph below. Larch species gain more tensile strength when soaked in water for extended periods. Cellulose and lignin, which are super-absorbent and super-retentive, are abundant in Larch species. Based on some literature studies [12,27], it has been found that soaking pine wood in low-salt water for two weeks can also reduce its lignin and hemicellulose content, increasing its susceptibility to impregnation in the future, and improving its resistance to external influences. Soaking pine wood in water for a short period can also reduce its hardness, making it easier to work with. A consistent comparison of tensile deformation behavior can be made using stress-normalized displacement responses in conjunction with observed failure mechanisms in the absence of direct strain measurements at local locations.

The present study used optical microscopy to assess structural changes induced by saline exposure; however, detailed microstructural examination of fracture zones (e.g., SEM analysis of failure surfaces) was beyond the scope of this study and should be addressed in future studies to complement the energy-based mechanical characterization proposed here. According to the results obtained for each investigated species, Cycle *III* was the most effective saline conditioning duration that provided the highest tensile strength and the most beneficial mechanical response for Scots pine and European larch, while Norway spruce demonstrated a progressive increase in mechanical resistance with conditioning duration and the best overall performance after Cycle *III*. Saline exposure improves stress transfer within wood structures, but its magnitude varies by species. A controlled saline conditioning method may improve wood reliability in environments with high humidity or salt exposure, such as coastal constructions, hydraulic structures, or outdoor structural elements, from an application perspective. In addition, the energy-based deformation analysis proposed in this study may be useful for selecting and predicting materials’ durability. The future should focus on detailed microstructural fracture analysis, quantification of salt penetration depth and transport mechanisms, environmental testing for long periods, and the extension of the energy-based methodology to engineered wood and other wood species. 

## 5. Conclusions

The study confirmed that saline conditioning affects softwood species differently, depending on moisture-related factors, internal structure, and deformation mechanisms. Based on these results, it is clear that tensile strength values alone cannot adequately describe this behavior. Results from tensile tests on pine, spruce, and larch showed that strength parameters did not always follow linear or monotonic trends over successive conditioning cycles. Wood, owing to its anisotropic nature, is susceptible to moisture-induced swelling and to stress redistribution, driven by its cell wall structure, which promotes microstructural rearrangements during loading. As a result, specimens with similar tensile strength values often exhibited markedly different deformation patterns. Despite the limitations of conventional strength-based assessments in evaluating wood materials subjected to environmental modifications, these findings underscore the need for complementary descriptors that capture the complete mechanical response.

The mechanical performance of the investigated materials is evaluated by considering the total tensile work, defined as the integral of stress–strain over the entire deformation process. Based on the integrated equations, clear species- and conditioning-duration-dependent differences in fracture development. Mechanical energy is absorbed and dissipated differently by different materials before failure, suggesting distinct mechanisms of deformation. In several cases, specimens with comparable maximum tensile loads had substantially different tensile work values, demonstrating that energy-based parameters are more sensitive to variations in deformation behavior than strength metrics alone. Under the applied loading conditions, the fitted integral functions exhibited high determination coefficients, confirming the robustness and reliability of the mathematical description.

By integrating tensile work analysis, a much more comprehensive interpretation of saline exposure’s effects on both load-bearing capacity and deformation energetics was possible. To predict the mechanical performance of wood materials, it is necessary to evaluate them under different environmental and service conditions. Compared with earlier investigations focused primarily on tensile-strength parameters of saline-treated wood [3,4], the present study demonstrates that energy-based descriptors derived from stress–strain relationships can effectively differentiate materials whose tensile strength is similar but whose deformation response is fundamentally different [3,4]. Among the studied saline conditioning procedures, Cycle *III* provided the best mechanical response and highest tensile strength for all investigated species.

## Figures and Tables

**Table 1:** Color parameters (L*, a*, b*) of the study material.

Material	L*	a*	b*	*G* Shine
European larch	76.50	5.90	25.44	3.20
Scots pine	61.01	10.86	25.47	1.90
Norway spruce	70.76	8.23	27.23	2.20

**Table 2:** Mean color changes (ΔE) and standard deviation (SD) of wood species depending on the saline treatment cycle.

Species	Cycle	ΔE (–)	SD
Pine	Native	0.00	–
Pine	*I*	2.34	0.41
Pine	*II*	4.87	0.66
Pine	*III*	6.12	0.73
Larch	Native	0.00	–
Larch	*I*	1.98	0.35
Larch	*II*	3.15	0.52
Larch	*III*	4.01	0.60
Spruce	Native	0.00	–
Spruce	*I*	2.10	0.39
Spruce	*II*	3.92	0.58
Spruce	*III*	5.45	0.69

**Table 3:** The maximum elasticity, normalized displacement, and yield stress values for pine wood samples.

Cycle	Apparent Stiffness Parameter, MPa	Determination Coefficient R^2^	Maximum Elasticity Displacement, mm	Yield Strength, MPa
Native	15.987	0.7865	5.090	59.703
*I*	38.997	0.9688	0.883	31.097
*II*	30.106	0.9873	0.970	26.973
*III*	48.055	0.9995	0.625	29.267

**Table 4:** The maximum elasticity, normalized displacement, and yield stress values for larch wood samples.

Cycle	Apparent Stiffness Parameter, MPa	Determination Coefficient R^2^	Maximum Elasticity Normalized Displacement, mm	Yield Strength, MPa
Native	17.814	0.8945	0.333	5.427
*I*	32.075	0.873	0.258	7.199
*II*	20.527	0.9148	18.901	2.808
*III*	46.473	0.9456	0.617	25.040

**Table 5:** The maximum elasticity, normalized displacement, and yield stress values for spruce wood samples.

Cycle	Apparent Stiffness Parameter, MPa	Determination Coefficient R^2^	Maximum Elasticity Normalized Displacement, mm	Yield Strength, MPa
Native	38.205	0.9688	1.058	38.284
*I*	27.520	0.8939	0.616	14.045
*II*	34.526	0.9464	1.225	42.030
*III*	46.710	0.9625	1.108	43.907

**Table 6:** The integral equation of total tensile work.

Total Work Carried Out Under Specified Conditions *W*_(*τ*,*φ*)_	Determination Coefficient R^2^	Normalized Displacement l, mm	Total Compaction Work, J
$W_{\left(Pine,Native\right)}=\int_{0}^{0.001\cdot l}(0.11x^{3}-2.81x^{2}+23.57x)dx$	0.9871	14.290	2.4 × 10^−3^
$W_{\left(Pine,Cycle\;I\right)}=\int_{0}^{0.001\cdot l}(-3\times 10^{-5}x^{6}+2.5\times 10^{-3}x^{5}-0.08x^{4}+1.3x^{3}-9.37x^{2}+31.38x)dx$	0.9277	25.275	9.97 × 10^−3^
$W_{\left(Pine,Cycle\;II\right)}=\int_{0}^{0.001\cdot l}(-1\times 10^{-4}x^{6}+6.6\times 10^{-4}x^{5}-0.17x^{4}+2.13x^{3}-12.7x^{2}+34x)dx$	0.901	22.483	8.5 × 10^−3^
$W_{\left(Pine,Cycle\;III\right)}=\int_{0}^{0.001\cdot l}(-2.53\times 10^{-2}x^{4}+0.83x^{3}-8.67x^{2}+34.6x)dx$	0.9061	13.580	3.18 × 10^−3^
$W_{\left(Larch,Native\right)}=\int_{0}^{0.001\cdot l}(1.1\times 10^{-3}x^{5}-0.04x^{4}+0.46x^{3}-2.37x^{2}+7.44x)dx$	0.9452	15.233	8.60 × 10^−4^
$W_{\left(Larch,Cycle\;I\right)}=\int_{0}^{0.001\cdot l}(-2.09x^{4}+14.69x^{3}-35.57x^{2}+36.01x)dx$	0.9904	3.325	1.99 × 10^−4^
$W_{\left(Larch,Cycle\;II\right)}=\int_{0}^{0.001\cdot l}(0.01x^{5}-0.31x^{4}+3.46x^{3}-16.49x^{2}+29.99x)dx$	0.9009	9.450	1.33 × 10^−3^
$W_{\left(Larch,Cycle\;III\;\right)}=\int_{0}^{0.001\cdot l}(0.09x^{5}-1.82x^{4}+13.08x^{3}-41.41x^{2}+57.41x)dx$	0.9305	7.408	1.57 × 10^−3^
$W_{\left(Spruce,Native\;\right)}=\int_{0}^{0.001\cdot l}(0.01x^{5}-0.19x^{4}+2.6x^{3}-15.762x^{2}+43.515x)dx$	0.9433	13.758	4.11 × 10^−3^
$W_{\left(Spruce,Cycle\;I\;\right)}=\int_{0}^{0.001\cdot l}(-6\times 10^{-5}x^{6}+3.5\times 10^{-3}x^{5}-8.8\times 10^{-2}x^{4}+1.09x^{3}-6.59x^{2}+18.37x)dx$	0.8701	19.667	3.54 × 10^−3^
$W_{\left(Spruce,Cycle\;II\;\right)}=\int_{0}^{0.001\cdot l}(13.26x^{2}+22.56x)dx$	0.9669	1.225	1.7 × 10^−5^
$W_{\left(Spruce,Cycle\;III\right)}=\int_{0}^{0.001\cdot l}(-0.1x^{4}+2.24x^{3}-17.13x^{2}+51.8x)dx$	0.9232	10.258	2.72 × 10^−3^

**Table 7:** Native material tensile tests along fibers.

Material	Cycle	Tensile Strength Along the Fibres, MPa (SD)
Pine	Native	72.46 (17.27) ^a,d^
	*I*	91.20 (2.80) ^d^
	*II*	39.71 (9.09) ^a,b,c^
	*III*	83.73 (18.31) ^d^
Larch	Native	20.73 (11.82) ^b,c^
	*I*	16.02 (10.61) ^b^
	*II*	18.91 (35.64) ^a,b,c^
	*III*	37.40 (9.24) ^a,b,c^
Spruce	Native	55.69 (32.37) ^a,c,d^
	*I*	60.94 (28.92) ^a,d^
	*II*	42.03 (32.00) ^a,b,c^
	*III*	62.43 (11.52) ^a,d^

SD—standard deviation, ^a,b,c,d^—homogeneous group.

## Data Availability

The original contributions presented in this study are included in the article. Further inquiries can be directed to the corresponding author.
